# Scrotal hemorrhage after testicular sperm aspiration may be associated with phosphodiesterase-5 inhibitor administration: a retrospective study

**DOI:** 10.1186/s12894-018-0316-9

**Published:** 2018-02-06

**Authors:** Yong-tong Zhu, Rui Hua, Song Quan, Wan-long Tan, Qing-jun Chu, Chun-yan Wang

**Affiliations:** 1 Reproductive Medicine Center, Department of Obstetrics and Gynecology, Nanfang Hospital/ The First School of Clinical Medicine, Southern Medical University, Guangzhou, China; 2 Department of Neurology, Integrated Hospital of Traditional Chinese Medicine, Southern Medical University, Guangzhou, China; 3 Department of Urology, Nanfang Hospital, Southern Medical University, Guangzhou, China

**Keywords:** Scrotal hemorrhage, Testicular sperm aspiration, Phosphodiesterase-5 inhibitor

## Abstract

**Background:**

Scrotal hemorrhage after testicular sperm aspiration (TESA) is uncommon in clinical operation. Phosphodiesterase-5 inhibitors (PDE5i) are commonly given to men who have difficulty providing a sperm sample for assisted reproductive technique such as in vitro fertilization. In this study, we examine the incidence of scrotal hemorrhage after TESA in men who received a PDE5i.

**Methods:**

In this retrospective study, 504 men with TESA operation in Center for Reproductive Medicine, Nanfang Hospital, Southern Medical University were collected. Men in the drug group had taken orally PDE5i before TESA. Men in the control group only operated TESA. The testis volume, coagulation function were measured. Sonographic examination with Doppler imaging was performed when scrotal hemorrhage appeared.

**Results:**

A total of 504 men with a mean age of 28.63 ± 4.22 years were included in the analysis. Of these, 428 did not receive a PDE5i prior to TESA and 76 received a PDE5i prior to TESA. Measures of coagulation function were not different between the groups. The incidence of hemorrhage was 0.0% in the control group and the drug group was 5.3%. The incidence of hemorrhage between two groups was different significantly (*P* = 0.000).

**Conclusion:**

In summary, the results of this study suggest that a PDE5i administration increases the risk of scrotal hemorrhage in men undergoing TESA, although the study design does not allow drawing a conclusion of cause and effect. Given the potential risk of scrotal hemorrhage after the ingestion of PDE5i, it may be wise not to administer it to men in whom a TESA may be performed.

## Background

Over the past several decades, in vitro fertilization and intracytoplasmic sperm injection (ICSI) have come into routine practice. On the day of oocyte retrieval, the male partner is typically asked to provide sperm by masturbation. However, a small number of males are able to provide sperm by masturbation due to psychologic stress and anxiety. These individuals are generally provided psychological guidance and administered a phosphodiesterase-5 inhibitor (PDE5i). When these measures are not successful, testicular sperm aspiration (TESA) is performed. Unfortunately, several males experienced scrotal hemorrhage after TESA.

As the worsening of the doctor-patient relationship in China, the violence in hospitals in China was growing and Chinese doctors were under tremendous stress [1]. It is necessary to look for the possible reason of scrotal hemorrhage after TESA and avoid such events. Surprisingly, none of azoospermia patients appeared scrotal hemorrhage after biopsy with the same TESA operation. It is assumed that scrotal hemorrhage after TESA may be associated with PDE5i administration. To our knowledge, reports about such cases are scarce. Therefore, this retrospective study was designed to summarize the data and answer this question.

## Methods

### Study design

We retrospectively reviewed the records of 504 men who underwent TESA at the Center for Reproductive Medicine, Nanfang Hospital, Guangzhou, China From 2012 to 2015. This study was approved by the Clinical Medical Local Ethical Review Committee of the Southern Medical University. Informed written consent was provided by all men in the study.

The men were classified into 2 groups; those who received a PDE5i before TESA and those who did not receive a PDE5i. Briefly, if men were unable to provide a sperm sample by masturbation due to stress/anxiety, and met the criteria described below, they were given tadalafil 20 mg and attempted to provide a sample by masturbation again. If a sample could not be provided TESA was performed. The time from tadalafil administration to TESA was approximately 3 h. The criteria of men in whom TESA was performed directly (not given the option of masturbation, control group) are described below.

Five hundred four men in this study were all infertility more than 1 year. Inclusion criteria for the drug group were: (1) had ejaculation by masturbation successfully more than twice, (2) were not able to ejaculation on the day of oocyte retrieval, (3) sperm were identified on semen analyses. Inclusion criteria for the control group were: (1) previously identified azoospermia, which contained obstruction azoospermia and non- obstruction azoospermia, or cryptozoospermia [2], (2) testis volumes were more than 8 mL. Patients in the drug group were all sperm retrieval performed by TESA successfully. In order to compare consistently, patients with prior epididymal sperm aspiration (PESA), testicular sperm extraction (TESE) or micro-TESE were excluded from this study.

### TESA procedure

**Fig. 1:**
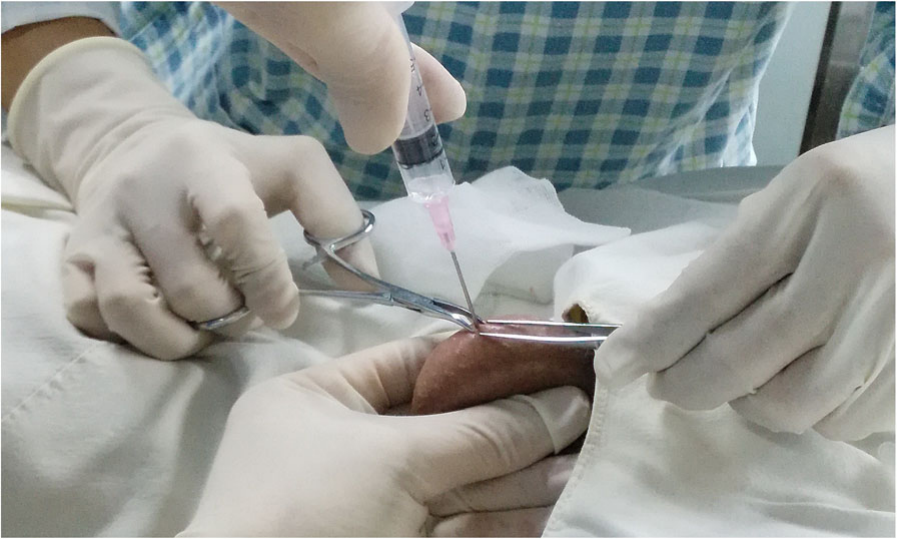
The testicular sperm aspiration procedure

The testis was anesthetized using 1% lidocaine. A 23 gauge needle was passed through the scrotal skin into the testicular tissue. Suction was applied with a 5 mL syringe, and the backpressure was maintained by hand. The needle was pushed in different directions into the testicular tissue to obtain a sample. Once an adequate sample was obtained, the needle was slowly removed from the testis while the negative pressure was maintained (Fig. 1). The sample was placed on a sterile plate, and the small tubules recovered were picked up by an assistant using 2 pairs of fine tweezers. Sperm in aspirated tissue was retrieved for ICSI or biopsy. All procedures were performed by Dr. Chu and Dr. Zhu.

### Follow-up

Patients were given wound care instructions, and instructed to keep the puncture site dry and clean for 3 days. They were asked about light level of activity following the procedures, and abstained from strenuous exercise for 1 month. Patients were seen for follow-up at 3 day, 1 week, and 1 month after TESA. At the follow-up visits, examination of the scrotum was performed for identification of hematoma formation. Patients were also asked the following 2 questions at each visit: (1) Did you avoid the strenuous exercise? (2) Did you feel uncomfortable in your scrotum?

Sonographic examination with Doppler imaging was performed if physical examination was consistent with a scrotal hemorrhage. If a hemorrhage did not self-absorb during 4- week period, an evacuation procedure was performed to remove the remaining blood products and clots.

### Statistical analysis

Calculations were analyzed by using SPSS 19.0 software (SPSS Inc., Chicago, Illinois, USA). All numeric data were presented as the mean value ± standard deviation. Frequencies were expressed as percentages. Students t-test was used for comparisons between 2 groups, and Fischer’s exact test was used for comparison of proportions. Values of *P* < 0.05 were considered to indicate statistically significant differences.

## Results

A total of 504 men who received TESA from 2012 to 2015 were included in the analysis. Mean age of the men was 28.63 ± 4.22 years. The testis size, character and the presence of testicular mass, or asymmetry was assessed via manual palpation and orchidometer. Mean testis volume was 11.8 ± 2.6 ml (Table 1). Coagulation function, which contained Thrombin time, Activated partial thromboplastin time, International normalized ratio, Prothrombin time and Fibrinogen were not different between the 2 groups. There were 428 men in the control group (no PDE5i) and 76 men in the drug group. The overall incidence of scrotal hemorrhage in the 504 men was 0.8%. However, the incidence in the control group was 0.0%, and in the PDE5i group was 5.3% (4 men) (*P* = 0.000). All 4 patients required an evacuation procedure. No other post-operative complications were noted.

**Table 1:** Characteristics of patients

	Control group	Drug group	Overall	*P* value
Number	428	76	504	
Age (years)	28.40 ± 3.86	29.07 ± 4.11	28.63 ± 4.22	*P* > 0.05
Testis volume (mL)	12.0 ± 4.3	11.5 ± 2.9	11.8 ± 3.6	*P* > 0.05
Coagulation function				
Thrombin time (sec)	15.24 ± 2.11	14.98 ± 2.03	15.11 ± 2.14	*P* > 0.05
Activated partial thromboplastin time (sec)	25.71 ± 3.23	26.01 ± 3.17	25.89 ± 3.26	*P* > 0.05
International normalized ratio	0.89 ± 0.23	0.91 ± 0.22	0.90 ± 0.22	*P* > 0.05
Prothrombin time (sec)	11.65 ± 1.09	12.02 ± 1.16	11.87 ± 1.18	*P* > 0.05
Fibrinogen (g/L)	2.55 ± 1.03	2.64 ± 1.06	2.62 ± 1.07	*P* > 0.05
Incidence of hemorrhage (%)	0(0.0)	4(5.3)	4(0.8)	*P* = 0.000

## Discussion

TESA, which was developed in 1992, is a method for retrieving sperm for use in assisted reproductive technology [3]. The procedure is also used to perform biopsy of the testis. Compared to TESE, TESA is a simpler procedure with minimal physiological consequences [4]. The majority of patients in the control group were obstructive patients. While azoospermic patients who had smaller testicular volume (< 8 mL), especially in the setting of testicular hypofunction, TESE or micro-TESE would be more appropriate in these patients.

It has been reported that intra-testicular hematoma formation occurs in 29% of diagnostic testicular biopsies [5]. However, scrotal hemorrhage was a relatively rare clinical event after TESA. In the current study of 504 TESA procedures, the incidence was only 0.8%. The difference in rates may be due to the increased use of sonographic examination. Most patients do not feel uncomfortable after TESA, and routine sonographic examination is not performed, and thus small areas of hemorrhage maybe overlooked.

At our institution, during the period from 2013 to 2015, 76 men successfully ejaculated by masturbation more than 2 times, but they were not able to ejaculation on the day of oocyte retrieval. Patients on intracavernosal injection treatment had high withdrawal rates. The most common reason for withdrawal was poor response to the therapy, followed by the inconvenience of use [6]. So they did not receive such therapy in our centre. These men passed through a procedure of relaxation, given pornographic material, mood adjusting and PDE5i drug taking, selected TESA operation finally to retrieve sperm. Although the proportion of these men who developed a scrotal hemorrhage was only 2.6%, no scrotal hemorrhage occured after same operation in the other 428 patients who did not receive a PDE5i. The results suggest that the scrotal hemorrhage in these 4 patients was related to the use of a PDE5i.

PDE5i, such as sildenafil (Viagra), vardenafil (Levitra) and tadalafil (Cialis), are used to treat erectile dysfunction. PDE5i increases nitrous oxide (NO) and cyclic guanosine monophosphate (cGMP) in the smooth muscles of the corpus cavernosum. For a PDE5i to be effective, sufficient sexual stimulation is essential [7]. The men who received a PDE5i still could not relax enough to achieve sexual arousal and could not successfully ejaculate. PDE5i are generally safe and well tolerated [8], have not been reported in association with scrotal hemorrhage events. As men were not able to ask to stop PDE5i before any surgical procedure, the rationale for excess bleeding in men taking PDE5i may not exist. Although our results showed a correlation between taking a PDE5i and scrotal hemorrhage, a cause-effect relationship could not be determined from the study design. However, the mechanism by which a PDE5i increases the risk of a scrotal hemorrhage may be as follows. First, a PDE5i results in vasodilation, and redistribution of arterial blood flow that is associated with rupture of vessels. Second, the NO and cGMP pathway might be responsible for inhibition of platelet aggregation and activation. Finally, PDE5i are considered as an antithrombotic agent [9].

The limitations of this study include its retrospective design. A prospective study should be done to validate our results. Although a large number of patients participated in this study, it appears as there were only 4 events (hematoma) in the 504 patients, which may be not enough to draw the conclusion and the results maybe only anecdotal. As ultrasound was only performed after the physician suspected a hematoma on physical exam post procedure. This may introduce significant bias and lack of certainty if there actually were many other patients that did not develop hematomas that were not detected by the clinician performing a 3 days post procedure exam. So it would be better to perform ultrasound exam at 3 days after TESA.

Although there were no sufficient evidences which could support that scrotal hemorrhage after TESA was caused by the administration of a PDE5i. Given the potential risk of scrotal hemorrhage after the ingestion of a PDE5i, it should be cautious to prescribe this medicine when the patient is likely to perform TESA. Patients were on anti-coagulants, anti-platelets, taking NSAIDs and ice scrotum may be beneficial after TESA. Although it is difficult to predict ahead who have difficulty producing a semen sample of the day of assisted reproductive technique, as all patients in the drug group had been able to produce ejaculated sperm twice prior to the procedure date. We should gain information by detailed inquiry. It would be better to prepare frozen sperm before the day of the retrieval once patient were categorized as masturbation difficulty males.

## Conclusion

In summary, the results of this study suggest that a PDE5i administration increases the risk of scrotal hemorrhage in men undergoing TESA, although the study design does not allow drawing a conclusion of cause and effect. Given the potential risk of scrotal hemorrhage after the ingestion of PDE5i, it may be wise not to administer it to men in whom a TESA may be performed.

## Abbreviations

cGMP: Cyclic guanosine monophosphate
ICSI: Intracytoplasmic sperm injection
NO: nitrous oxide
PDE5i: Phosphodiesterase type5 inhibitor
PESA: Epididymal sperm aspiration
TESA: Testicular sperm aspiration
TESE: Testicular sperm extraction
